# Insulin-Resistant Diabetes Associated With a Novel POLD1 Variant of Uncertain Significance in a Pediatric Patient

**DOI:** 10.7759/cureus.111260

**Published:** 2026-06-21

**Authors:** Somaya K Alzelaye, Fuad M Alkudaysi, Ali Alghanmi, Turki Alhasani

**Affiliations:** 1 Pediatrics, Endocrine and Diabetic Center, Al-Qunfudah General Hospital, Al-Qunfudah, SAU; 2 Pediatrics, South Al-Qunfudah Hospital, Al-Qunfudah, SAU; 3 Pediatrics, Khamis Mushayt Maternity and Children Hospital, Khamis Mushayt, SAU

**Keywords:** acanthosis nigricans, insulin resistance, lipodystrophy, mdpl syndrome, pediatric diabetes, pold1, variant of uncertain significance

## Abstract

Mandibular hypoplasia, deafness, progeroid features, and lipodystrophy (MDPL) syndrome is a rare autosomal dominant disorder caused by pathogenic variants in the *POLD1 *gene and characterized by progressive lipodystrophy and severe metabolic complications. We report an eight-year-old Saudi male presenting with atypical, insulin-resistant diabetes, acanthosis nigricans, and preserved C-peptide levels, raising suspicion for a syndromic form of diabetes. Genetic testing revealed a heterozygous variant of uncertain significance (VUS) in *POLD1*.

Notably, the patient lacked classical features of MDPL, including overt lipodystrophy, mandibular hypoplasia, and hearing loss, posing a diagnostic challenge. Although the patient’s phenotype showed partial overlap with reported *POLD1*-related disorders, the available evidence was insufficient to establish a definitive molecular diagnosis.

Early recognition of atypical presentations of severe insulin resistance is crucial for appropriate evaluation, surveillance, and genetic counseling. Further functional studies and long-term follow-up are required to clarify the pathogenicity of the identified variant and its clinical implications.

## Introduction

Lipodystrophy syndromes are a heterogeneous group of disorders characterized by partial or generalized loss of adipose tissue, often leading to significant metabolic complications such as insulin resistance, diabetes mellitus, hypertriglyceridemia, and hepatic steatosis [1]. Among these, autosomal dominant partial lipodystrophies are frequently associated with additional systemic and structural features beyond metabolic abnormalities.

Mandibular hypoplasia, deafness, progeroid features, and lipodystrophy (MDPL) syndrome (OMIM #615381) is an ultra-rare form of autosomal dominant partial lipodystrophy that was first described in 2010. The condition is typically recognized by a combination of progressive loss of subcutaneous fat, usually becoming more apparent after puberty, sensorineural hearing loss, characteristic craniofacial features (including mandibular hypoplasia and a beaked nose), and features suggestive of premature aging. Musculoskeletal involvement, such as joint stiffness and contractures, has also been reported [2].

In 2013, pathogenic variants in the *POLD1 *gene, which encodes the catalytic subunit of DNA polymerase δ, were identified as the underlying cause of MDPL syndrome. These findings linked MDPL to a broader group of disorders related to impaired DNA replication and repair. Dysfunction of the exonuclease proofreading domain of polymerase δ is thought to contribute to genomic instability, providing a possible explanation for both the metabolic and progeroid manifestations of the disease [3].

Since its molecular characterization, only a limited number of genetically confirmed MDPL cases - estimated at around 30-40 - have been reported in the literature. Although the core clinical features are relatively consistent, there appears to be variability in the severity and timing of presentation [4].

Progressive lipodystrophy, mandibular hypoplasia, and hearing loss are considered key features, but the extent of metabolic involvement can vary between patients. Many affected individuals develop severe insulin resistance, early-onset diabetes, and hypertriglyceridemia, which in some cases may lead to complications such as pancreatitis [3]. The pattern of fat loss in MDPL also appears distinct from other forms of familial partial lipodystrophy, with relative preservation of adipose tissue in the neck and supraclavicular regions [2].

Most pathogenic *POLD1 *variants reported in MDPL are de novo and cluster within the exonuclease domain, which is critical for polymerase proofreading activity [3]. The most commonly reported variant is p.Ser605del, although additional missense and in-frame variants have also been described [3,4]. Functional studies of several variants demonstrated impaired exonuclease activity and increased cellular dysfunction, supporting the proposed relationship between *POLD1 *abnormalities and the clinical manifestations observed in MDPL syndrome [3,4].

Despite these advances, important aspects of MDPL syndrome remain unclear, particularly in relation to its full phenotypic spectrum, natural history, and optimal management strategies. In addition, the clinical significance of newly identified *POLD1 *variants-especially those classified as variants of uncertain significance-can be difficult to determine in the absence of functional or familial data.

In this report, we describe a pediatric patient with an atypical form of insulin-resistant diabetes in whom a heterozygous *POLD1 *variant was identified. The clinical presentation does not fully match the classical description of MDPL, raising questions about the significance of this finding. Through this case, we aim to highlight the challenges involved in interpreting genetic results in rare disorders and to contribute to the growing understanding of the potential variability in *POLD1*-related disease.

## Case presentation

An eight-year-five-month-old Saudi Arabian male was initially referred to the pediatric endocrinology clinic at age 7 for the evaluation of newly diagnosed diabetes mellitus of three months' duration. The presenting history was notably atypical for classic autoimmune type 1 diabetes (T1DM), with an absence of polyuria, polydipsia, or significant weight loss. His past medical history is significant for learning difficulties, behavioral abnormalities, and carious teeth. There is no family history of a similar metabolic disorder; the consanguinity status of the parents is unknown.

Physical examination revealed a key dermatologic finding: generalized acanthosis nigricans on the neck, a hallmark of severe insulin resistance. A cardiac murmur was also auscultated. Initial laboratory workup is shown in Table 1.

**Table 1:** Summary of laboratory investigations RBC: Red blood cells; Hct: Hematocrit; MCV: Mean corpuscular volume; MCH: Mean corpuscular hemoglobin; MCHC: Mean corpuscular hemoglobin concentration; RDW: Red cell distribution width; WBC: White blood cells; AST: Aspartate aminotransferase; ALT: Alanine aminotransferase; LDL: Low-density lipoprotein; HDL: High-density lipoprotein.

Test	Result	Unit	Reference range
Hematology			
Hemoglobin (Hb)	137	g/L	115–155
RBC count	5.42	×10¹²/L	4.0–5.2
Hematocrit (Hct)	0.43	L/L	0.35–0.45
MCV	79.5	fL	75–87
MCH	25.3	pg	24–30
MCHC	318	g/L	320–360
RDW	13.9	%	11.5–14.5
Platelets	304	×10⁹/L	150–450
MPV	10.9	fL	7–12
WBC	4.02	×10⁹/L	4.5–13
Neutrophils	30.9	%	40–60
Lymphocytes	56	%	30–50
Monocytes	11.4	%	2–8
Eosinophils	1	%	1–4
Basophils	0.7	%	0–1
Liver function			
AST	28	U/L	10–40
ALT	15	U/L	7–56
Albumin	42	g/L	35–50
Total bilirubin	7.4	µmol/L	3–20
Direct bilirubin	2	µmol/L	0–5
Lipid profile			
Total cholesterol	4.74	mmol/L	<5.2
LDL	2.4	mmol/L	<3.4
HDL	2.0	mmol/L	>1.0
Triglycerides	0.7	mmol/L	<1.7
Bone and mineral metabolism			
Alkaline phosphatase	218	U/L	150–420
Phosphorus	1.51	mmol/L	1.3–2.3
Calcium	2.51	mmol/L	2.2–2.6
Renal function			
Urea nitrogen	2.8	mmol/L	2.5–6.5
Creatinine	17.7	µmol/L	20–60
Diabetes workup			
HbA1c	9.4	%	<5.7
C-peptide	0.7	ng/mL	0.5–2.0
Insulin	1030	pmol/L	17–173

The patient was commenced on a basal-bolus insulin regimen (insulin glargine and aspart). Over approximately two years of follow-up, his insulin requirements remained modest but gradually increased (from 8 to 10 units of basal insulin), with persistent suboptimal glycemic control. The patient was not adherent to self-monitoring of blood glucose.

Given the phenotypic clues of insulin resistance and the clinical suspicion of a syndromic form of diabetes, a genetic analysis was performed. This test identified a heterozygous variant of uncertain significance (VUS) in the *POLD1 *gene. The identified variant was *POLD1 *c.2643-26A>C, a novel intronic noncoding variant classified as a VUS (ACMG Class 3). No functional studies, segregation data, or prior reports linking this specific variant to disease were available at the time of evaluation.

The patient has atypical insulin-resistant diabetes associated with a novel *POLD1 *VUS. While certain clinical findings raise suspicion for a possible *POLD1*-related disorder, current evidence is insufficient to establish a diagnosis of MDPL syndrome. ACMG/AMP (American College of Medical Genetics and Genomics/the Association for Molecular Pathology) interpretation of the identified *POLD1 *variant is shown in Table 2.

**Table 2:** ACMG/AMP interpretation of the identified POLD1 variant Variant classification was performed according to ACMG/AMP guidelines. The *POLD1 *c.2643-26A>C variant is classified as a variant of uncertain significance (Class 3) due to limited available evidence, including absence from population databases (PM2_supporting) and lack of functional, segregation, or previously reported data. ACMG: American College of Medical Genetics and Genomics; AMP: Association for Molecular Pathology.

Criterion	Evidence	Status
Variant	*POLD1 *c.2643-26A>C	—
Variant type	Intronic noncoding variant	—
Population frequency	Absent from gnomAD/ESP/1000G	PM2_supporting
Functional studies	Not available	Not met
Segregation data	Not available	Not met
Previous reports	None identified	Not met
Computational evidence	Not available	Not met
ACMG classification	Variant of uncertain significance	Class 3

These findings - diabetes with acanthosis nigricans, preserved C-peptide, and absence of diabetic ketoacidosis - prompted a differential diagnosis extending beyond typical T1DM to include severe insulin resistance syndromes. Differential diagnostic considerations are provided in Table 3.

**Table 3:** Differential diagnostic considerations MDPL: Mandibular hypoplasia, deafness, progeroid features, and lipodystrophy; DKA: Diabetic ketoacidosis; INSR: Insulin receptor; VUS: Variant of uncertain significance.

Disorder	Supporting features	Findings against diagnosis
Type 1 diabetes	Childhood-onset diabetes	Preserved C-peptide and absence of DKA
Type 2 diabetes	Insulin resistance and acanthosis nigricans	Young age and syndromic concerns
Rabson-Mendenhall syndrome	Severe insulin resistance	No characteristic phenotype and no pathogenic INSR variant detected
Partial lipodystrophy	Insulin resistance	No documented lipodystrophy
MDPL syndrome	*POLD1 *VUS identified	Incomplete phenotype and VUS only

A summary of the key clinical events from initial presentation to current follow-up is provided in Table 4.

**Table 4:** Clinical timeline of disease progression and diagnostic evaluation DKA: Diabetic ketoacidosis; VUS: Variant of uncertain significance; MDPL: Mandibular hypoplasia, deafness, progeroid features, and lipodystrophy.

Age/Timepoint	Clinical event
Age: 7 years	Diagnosis of diabetes mellitus without typical symptoms
+3 months	Referral to a pediatric endocrinology clinic
Initial assessment	Acanthosis nigricans, preserved C-peptide, and no DKA
Early management	Initiation of basal-bolus insulin therapy
Follow-up (~2 years)	Persistent suboptimal glycemic control (HbA1c: 7.0%–8.5%)
During evaluation	Suspicion of severe insulin resistance syndrome
Genetic testing	Identification of heterozygous *POLD1 *VUS
Current status	Ongoing follow-up with incomplete MDPL phenotype

## Discussion

This case describes an eight-year-old Saudi male presenting with an atypical form of insulin-resistant diabetes, acanthosis nigricans, neurodevelopmental concerns, and a heterozygous VUS in the *POLD1 *gene. The clinical picture initially raised suspicion for severe insulin resistance syndromes such as Rabson-Mendenhall syndrome or partial lipodystrophy. The identification of a *POLD1 *variant added another layer of complexity, given the known association between *POLD1 *and MDPL syndrome, but without a fully consistent phenotype.

The patient’s presentation overlaps only partially with the features typically reported in MDPL syndrome. Severe insulin resistance and diabetes mellitus are consistent with previously reported cases, whereas overt lipodystrophy, craniofacial abnormalities, hearing loss, and clear progeroid features were not identified [2,3]. One possible explanation is that the patient represents an early or attenuated phenotype, particularly because lipodystrophy in MDPL often becomes more apparent after puberty [2]. Alternatively, the identified variant may not be fully pathogenic.

The presence of acanthosis nigricans and preserved C-peptide supports a state of significant insulin resistance, which aligns with the metabolic profile seen in lipodystrophy syndromes [1]. In such conditions, reduced functional adipose tissue leads to ectopic fat deposition and impaired insulin sensitivity. The patient’s learning and behavioral difficulties may also fit within the broader, though inconsistently reported, neurodevelopmental spectrum of *POLD1*-related disorders [5]. However, these findings are nonspecific and should be interpreted with caution.

Interpreting a VUS in this context remains challenging. Most pathogenic *POLD1 *variants reported in MDPL are de novo and affect the exonuclease domain, leading to impaired proofreading activity and genomic instability [3,5]. In our case, the absence of functional validation and parental testing limits the ability to determine the clinical significance of the variant. This is a common issue in current genomic practice, where genetic findings may outpace our ability to interpret them with confidence.

Management of this patient has primarily followed standard approaches for insulin-requiring diabetes, as no disease-specific therapies currently exist for *POLD1*-related disorders. Nevertheless, establishing a definitive molecular diagnosis would carry important implications for long-term surveillance and family counseling. Confirmed MDPL syndrome would warrant monitoring for metabolic complications, cardiovascular risk, hepatic involvement, skeletal abnormalities, and hearing impairment [2]. Genetic counseling would also be important, particularly in determining whether the variant occurred de novo.

Overall, this case illustrates the diagnostic uncertainty that can arise when genetic findings and clinical features do not fully align. It also suggests that the spectrum of *POLD1*-related disease may be broader than previously described, potentially including milder or earlier presentations. Careful longitudinal follow-up, combined with further genetic and functional studies, will be important in clarifying the significance of this variant and its role in the patient’s clinical phenotype.

Structured approach for future clinical and research endeavors

Based on the findings and challenges presented in this case, we recommend a structured approach for future clinical and research endeavors:

Comprehensive Phenotyping

We recommend a systematic evaluation for subtle signs of lipodystrophy (e.g., DEXA (dual-energy x-ray absorptiometry) scan and MRI for adipose tissue distribution), audiological testing, craniofacial imaging, and detailed dermatological and musculoskeletal assessments to seek evidence of an evolving MDPL phenotype.

Familial Segregation Analysis

Performing targeted genetic testing of the parents is imperative. Identifying the variant as de novo would strongly support its pathogenicity, whereas its inheritance from an unaffected parent would argue against a fully penetrant, classic MDPL-causing role.

Functional Studies

Where possible, collaborative efforts should be made to characterize the variant’s impact on DNA polymerase δ exonuclease activity in vitro, providing critical evidence to reclassify the VUS.

Longitudinal Follow-Up

Continued monitoring of this patient is essential to document the natural history, particularly through adolescence, to see if classic MDPL features emerge.

A major limitation of this report is the uncertain clinical significance of the identified *POLD1 *variant. The detected variant is a novel intronic alteration classified as a VUS. No functional studies, segregation analysis, or prior reports are currently available. Furthermore, several cardinal features of MDPL syndrome were absent. Therefore, a causal relationship between the identified variant and the patient's phenotype cannot currently be established.

## Conclusions

In conclusion, this case raises suspicion for a possible *POLD1*-related disorder in a child with atypical insulin-resistant diabetes. However, the identified variant remains of uncertain significance, and additional phenotyping, segregation studies, and functional validation are required before a causal relationship can be established. This case delineates the clinical challenges and underscores the need for an integrated diagnostic framework combining genetics, deep phenotyping, and functional assays to achieve precision medicine for patients with such rare disorders.
